# Identification of Key Genetic Markers for Drug Pseudoallergy via Experimental Transcriptomics and GWAS-Based Causal Inference

**DOI:** 10.3390/cimb48101000

**Published:** 2026-09-29

**Authors:** Lihui Zhang, Fangliang He, Jiating Zhang, Yuechen Zhao, Yifan Guo, Lizhi Wan, Jia Chen, Yongqiang Lin, Xianlong Cheng, Feng Wei

**Affiliations:** 1 Institute for Control of Traditional Chinese Medicine and Ethnic Medicine, National Institutes for Drug Control, Beijing 102629, China; zhanglihui@nifdc.org.cn (L.Z.);; 2 Chinese Academy of Medical Sciences & Peking Union Medical College, Beijing 100050, China; 3 Traditional Chinese Materia Medica, Shenyang Pharmaceutical University School, Shenyang 100730, China

**Keywords:** pseudoallergy, mast cells, RNA sequencing, gene signature, GWAS, Mendelian Randomization

## Abstract

Drug-induced pseudoallergic reactions are rapid in onset and complex in mechanism, with current clinical safety assessments lacking precise molecular underpinnings. Cells were treated with pseudoallergen-positive compounds. Cell degranulation was verified by neutral red staining and ELISA, followed by high-throughput RNA sequencing. Key signaling pathways were elucidated through Gene Ontology and Kyoto Encyclopedia of Genes and Genomes enrichment analyses. A pseudoallergic characteristic gene network was identified and constructed using WGCNA. Finally, two-sample MR analysis was performed utilizing GWAS data to evaluate the causal relationship between candidate genes and drug pseudoallergy, thereby identifying key genetic risk markers. The pseudoallergen-positive compounds significantly induced cell degranulation and extensive transcriptional regulation. Enrichment analyses indicated that MAPK, JAK-STAT, TNF, and PI3K-Akt signaling pathways play central roles in pseudoallergic reactions. WGCNA identified a pseudoallergic characteristic gene signature. Subsequent MR analysis identified EMP1 and KIF26A might be the key genetic risk markers associated with drug pseudoallergy. These two genes potentially increase the causal risk of drug-induced pseudoallergy by modulating membrane coating, vesicle formation, and related cellular transport processes. This study successfully established a mast cell characteristic gene signature for pseudoallergic reactions, elucidated the core transcriptional network, and identified key risk markers that may have a causal association with drug pseudoallergy.

## 1. Introduction

Drug-induced acute hypersensitivity reactions are common adverse drug reactions, characterized by rapid onset and potentially life-threatening outcomes; thus, their safety risks cannot be overlooked [1,2]. Acute hypersensitivity reactions are primarily categorized into IgE-mediated Type I hypersensitivity and non-IgE-mediated pseudoallergic reactions (or anaphylactoid). While clinically similar, their underlying mechanisms are distinct [3]. Pseudoallergic reactions are characterized by their non-sensitized nature, wherein allergic-like symptoms can be triggered upon first exposure to the causative agent. Their mechanisms are complex and diverse, primarily involving direct stimulation of mast cells or basophils by drugs to release bioactive mediators such as histamine and tryptase, as well as indirect induction of mast cell mediator release through activation of the complement [4] or kallikrein–kinin systems [5]. Notably, patients with mastocytosis exhibit a significantly elevated risk of severe drug-induced anaphylactic shock, further highlighting the central role of mast cells in pseudoallergic reactions [6].

Currently, a wide variety of drugs are known to induce pseudoallergic reactions, and the underlying sensitizing substances may include active pharmaceutical ingredients, pharmaceutical excipients, and other non-active components. This suggests that different drugs may trigger pseudoallergic reactions via diverse molecular pathways and targets [7,8]. It is widely accepted that upon entering the body, small molecule drugs primarily induce pseudoallergy by directly binding to specific receptors on the surface of mast cells and basophils, leading to their degranulation [9,10]. A broad spectrum of chemically diverse agents—ranging from synthetic compounds like mivacurium [11] to complex herbal mixtures (e.g., traditional Chinese medicine injections) and natural compounds—have been shown to trigger mast cell degranulation [12,13]. This chemical diversity suggests that while the initial triggers vary, they may converge on shared downstream intracellular signaling cascades. Identifying these common transcriptional responses is therefore essential for developing universal screening tools.

Given that different drugs may induce mast cell activation through diverse targets and mechanisms, leading to unique downstream signaling pathway effects, a deep elucidation of these molecular events is crucial for understanding the pathogenesis of pseudoallergic reactions and for drug risk assessment. This study aimed to systematically analyze gene expression profile data from MRGPRX2-transfected human mast cell H_MRGX2_HMC-1 and rat basophilic leukemia cell H_MRGX2_RBL-2H3 after intervention with various pseudoallergen-positive compounds (C48-80, PAMP-12, Substance P, and (R)-ZINC-3573) using high-throughput RNA sequencing (RNA-seq). By integrating advanced bioinformatics and computational biology analyses, this study will identify differential pathways and construct a characteristic gene signature for pseudoallergy. Furthermore, to bridge the gap between transcriptomic correlation and genetic causality, we performed a two-sample Mendelian randomization (MR) analysis. Using cis-expression quantitative trait loci (cis-eQTLs) of the identified signature genes as instrumental variables and drug pseudoallergy genome-wide association study (GWAS) data as outcomes, we characterized the key causal genetic markers underlying drug pseudoallergy. Our results provide precise molecular insights and a reliable evaluation strategy for the preclinical safety assessment of clinical medications.

## 2. Materials and Methods

### 2.1. Main Reagents and Instruments

The human mast cell line HMC-1 (YS1791C) was purchased from Yaji Biotechnology Co., Ltd. (Yaji Cell Center, Shanghai, China). Rat basophilic leukemia (RBL-2H3) cells (CL-0192) were obtained from Procell Life Science & Technology Co., Ltd., Wuhan, China. H_MRGX2_HMC-1 cells and H_MRGX2_RBL-2H3 cells were generated in our laboratory. Compound 48/80 (C48-80) (HY-115768), PAMP-12 (HY-P3419), Substance P (HY-P0201), and (R)-ZINC-3573 (HY-118069) were purchased from MCE Corporation. TRIzol Reagent (Cat. No. 15596018CN) for RNA extraction was purchased from Thermo Fisher Scientific. A NanoDrop 2000 spectrophotometer (Thermo Fisher Scientific, Waltham, MA, USA), Agilent 5300 Bioanalyzer (Agilent, Santa Clara, CA, USA), Qubit 4.0 fluorometer (Thermo Fisher Scientific), T100 Thermal Cycler PCR instrument (Bio-Rad, Hercules, CA, USA), and NovaSeq X Plus sequencer (Illumina, San Diego, CA, USA) were used.

### 2.2. Cell Culture

Both cell lines were cultured in a humidified incubator at 37 °C with 5% CO_2_. H_MRGX2_RBL-2H3 cells were maintained in MEM (Gibco, New York, NY, USA, Cat. No.: 11095080) supplemented with 10% fetal bovine serum (FBS; Gibco, Cat. No.: A5256701). H_MRGX2_HMC-1 cells were cultured in IMDM (Gibco, Cat. No.: 12440053) with 10% FBS. Adherent cells were passaged using 0.25% trypsin (Gibco, Cat. No.: 25200056) during their logarithmic growth phase. Cells between passages 3 and 10 were used for experiments.

### 2.3. Neutral Red Staining Assay

H_MRGX2_RBL-2H3 cells were seeded into 24-well plates. After grouped drug treatment, the supernatant was removed, and cells were stained with neutral red Stain (1:3000 dilution) for 5 min. Cells were then thoroughly washed with PBS, and observations and imaging were performed.

### 2.4. ELISA

As per the manufacturer’s protocol, supernatants from H_MRGX2_HMC-1 and H_MRGX2_RBL-2H3 cells treated with 20 μM of the compounds for 1 h were analyzed by ELISA. Each sample was tested in quintuplicate. The absorbance at 450 nm was measured on a microplate reader within 5 min, and tryptase and β-hexosaminidase concentrations were determined via standard curves.

### 2.5. RNA Extraction, Library Preparation, and Sequencing

A total of 36 cell samples were prepared from two cell lines. Each cell line consisted of 18 samples, which were divided into a normal control group (*n* = 6) and four compound treatment groups (C48-80, PAMP-12, Substance P, and (R)-ZINC-3573; all treated at 20 μM for 1 h, *n* = 3 per group). Total RNA was extracted from cells. RNA concentration and purity were measured, and integrity was assessed by agarose gel electrophoresis. mRNA was enriched using oligo-dT beads for transcriptome analysis. mRNA was fragmented, reverse transcribed into cDNA, ligated with adapters, purified, and size-selected. Libraries were amplified by PCR and purified. Paired-end sequencing of the libraries was performed on an Illumina NovaSeq X Plus platform. Raw sequencing reads were initially quality-assessed using FastQC (v0.11.9) and subsequently processed. Adapter sequences and low-quality bases (with a quality score Q < 30) were removed using fastp (v0.23.2) to obtain high-quality clean reads. These clean reads were then aligned to the respective reference genomes using HISAT2 (v2.2.1). For Homo sapiens, the GRCh38 (GCA_000001405.15) reference genome was used, and for the rat genome, GRCr8 (GCA_000001895.4). The corresponding gene annotation files for both genomes were downloaded from the Ensembl database (http://www.ensembl.org) (accessed on 26 November 2025). Finally, gene expression levels were quantified using featureCounts software (v2.0.3) against these Ensembl gene annotations.

### 2.6. Differential Gene and Enrichment Analysis

Differential expression gene (DEG) analysis was performed between the drug-treated groups (C48-80, PAMP-12, Substance P, and (R)-ZINC-3573, all at a final concentration of 20 μM for 1 h) and the normal control group using the edgeR package (v3.14.0) in R statistical software. The edgeR pipeline involved TMM normalization, filtering of low-count genes (retaining genes with a count-per-million (cpm) > 1 in at least 3 samples), and dispersion estimation using the estimateDisp function. A design matrix was constructed to model the effect of compound treatment, and differential expression was determined using generalized linear models (GLM) with exact testing. Prior to DEG analysis, principal component analysis (PCA) was performed to assess sample clustering and identify potential batch effects, confirming clear separation between control and treated groups and tight clustering of replicates. Genes with |log2FC| > 1 and adjusted *p*-value < 0.05 were considered significantly differentially expressed and selected for subsequent analysis. Gene Ontology (GO) functional annotation and Kyoto Encyclopedia of Genes and Genomes (KEGG) pathway enrichment analysis of DEGs were conducted using the clusterProfiler package (v4.16.0) in R. A *p*-value < 0.05 and a multiple testing-corrected false discovery rate (FDR) were used as significance thresholds.

### 2.7. Characteristic Gene Signature Construction

Co-expression modules were identified using the weighted gene co-expression network analysis (WGCNA) package (version 1.74) with RNA-seq data from H_MRGX2_HMC-1 cells. This strategic decision was made to focus on a high-confidence human-specific gene signature, minimizing potential confounding from species-specific transcriptional differences and ortholog mapping complexities inherent in cross-species analysis. An appropriate soft threshold of 6 (yielding a scale-free topology fit index R^2^ = 0.85) was set to construct a scale-free network, and the adjacency matrix between correlated genes was transformed into a topological overlap matrix (TOM). Modules were defined based on hierarchical clustering using networkType = “signed”, deepSplit = 2, mergeCutHeight = 0.25, and minModuleSize = 100 to ensure robust module detection. Pearson correlation coefficients between module eigengenes were calculated to assess module similarity. The union of genes from significantly correlated modules was taken. Hub genes were identified using the cytoHubba plugin with the MCC algorithm, and the network was visualized using Cytoscape v3.9.0.

### 2.8. Data Sources for Mendelian Randomization

MR analysis was conducted using GWAS data to investigate the potential causal relationship between genetic variants and drug-induced allergic reactions. Exposure data were obtained from cis-eQTLs provided by eQTLGen (https://eqtlgen.org/cis-eqtls.html) (accessed on 2 December 2025). Outcome GWAS data were retrieved from the OpenGWAS database (https://opengwas.io) (accessed on 2 February 2026), comprising a total of 1,284,690 samples (Table 1). Crucially, both exposure and outcome GWAS datasets were exclusively derived from individuals of European ancestry to minimize confounding due to population stratification. As this study utilized publicly available GWAS summary statistics for MR analysis, no additional ethical approval or informed consent was required.

### 2.9. Mendelian Randomization Analysis

For the identified genes with potential causal associations, a Benjamini–Hochberg correction was applied to control for multiple testing, with a corrected *p*-value < 0.05 considered significant. Steiger directionality testing was performed to ensure the correct causal direction from gene expression to outcome. Using the aforementioned criteria, a two-sample MR analysis was performed for the exposure and outcome using the ‘TwoSampleMR’ and ‘VariantAnnotation’ packages in R. Five analytical methods were employed: inverse-variance weighted (IVW), MR-Egger, weighted median, weighted mode, and simple mode. The IVW method was designated as the primary approach for estimating causal effects due to its statistical power, while the other four methods served to validate the robustness and consistency of the findings, particularly in the presence of pleiotropy.

Single nucleotide polymorphisms (SNPs) were selected based on a genome-wide significance level of *p* < 5 × 10^−8^ and an F-statistic ≥ 10, ensuring strong association with the exposure and minimizing weak instrument bias. To obtain independent SNPs and retain those most strongly associated with gene expression levels for subsequent analysis, a clumping threshold of r^2^ < 0.001 within a 10,000 base-pair (bp) physical distance was applied.

### 2.10. Sensitivity Analysis

Cochran’s Q test was utilized to detect heterogeneity among the individual SNP effects, which might indicate the presence of pleiotropy. The MR-Egger intercept test was employed to assess for directional pleiotropy, and leave-one-out analysis was conducted to identify influential SNPs and evaluate the consistency of the findings when each SNP was sequentially removed. Genes demonstrating a statistically significant association (*p* < 0.05) were categorized into high-risk genes (odds ratio [OR] > 1.0) and protective genes (OR < 1.0) based on their estimated OR values. Forest plots of strongly associated genes were generated based on the causal effect estimates from the IVW and weighted median analyses, utilizing the ‘grid’, ‘readr’, and ‘forestploter’ packages. Intersection analysis between identified risk genes and DEGs from parallel transcriptomic studies was performed using the ‘VennDiagram’ package, and the results were visualized as a Venn diagram.

### 2.11. Gene Set Enrichment Analysis (GSEA)

GSEA was performed using the R ‘clusterProfiler’ package (v4.16.0). Genes were ranked based on their log2 fold-change values derived from the differential expression analysis between pseudoallergen-treated and control groups. The ‘c2.cp.kegg.Hs.symbols.gmt’ and ‘c5.go.bp.v2023.2.hs.symbols.gmt’ gene set files from the MSigDB database (https://www.gsea-msigdb.org/gsea/) (accessed on 7 January 2026) were used. The number of permutations was set to 1000, and gene sets with a minimum size of 15 and a maximum size of 500 were considered. Normalized enrichment scores (NES) > 1, *p*-value < 0.05, and an FDR < 0.25 were used as significance thresholds.

## 3. Results

### 3.1. Compound-Induced Mast Cell Degranulation

**Figure 1:**
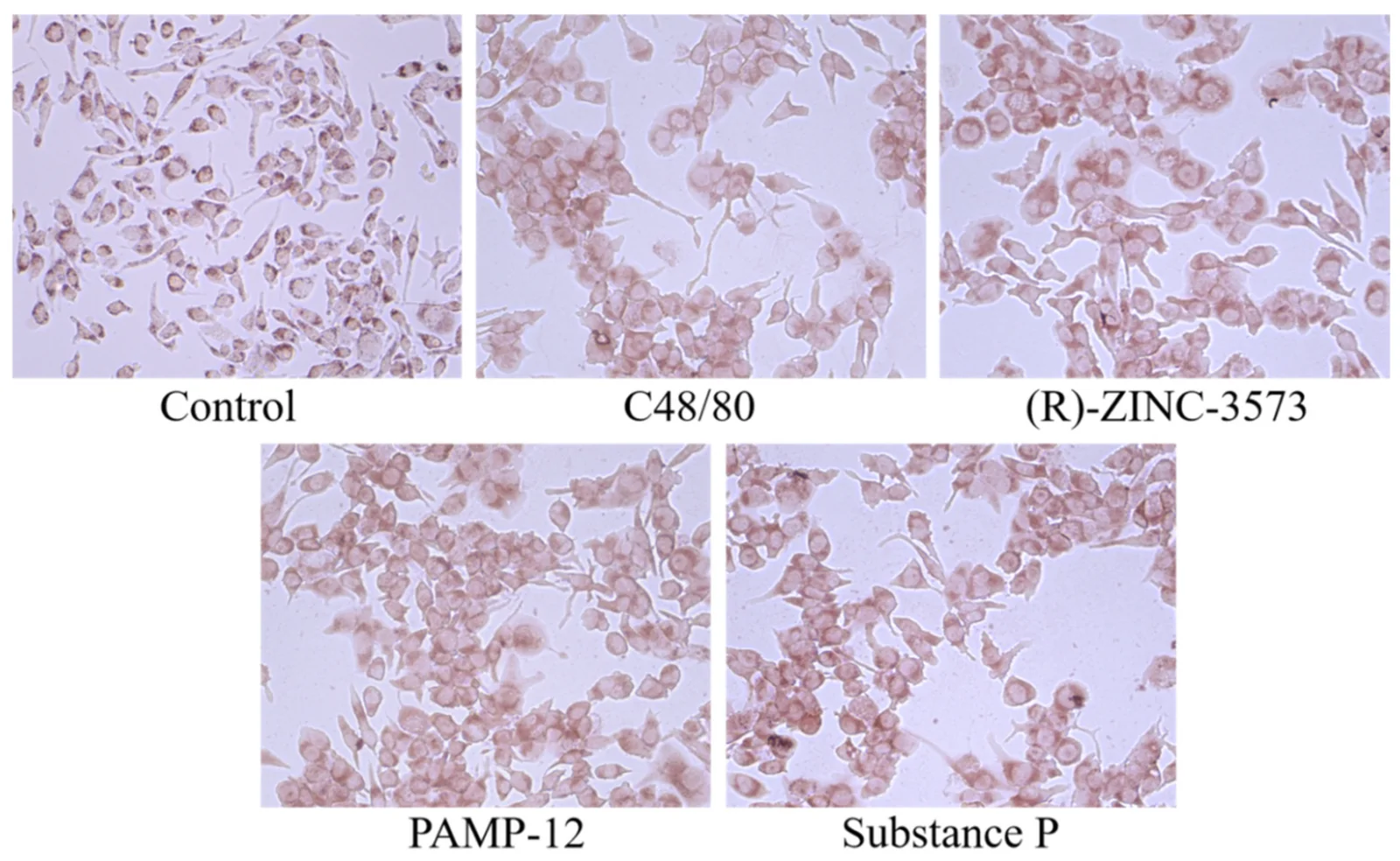
Microscopic morphology of H_MRGX2_RBL-2H3 cells stained with neutral red (200×).

Neutral red is a weakly basic, cationic, lipophilic dye that freely penetrates cell membranes. In unstimulated cells, neutral red accumulates in highly acidic cytoplasmic granules, appearing as clear red or reddish-orange puncta. Upon cell degranulation, granule contents (e.g., tryptase, histamine, β-hexosaminidase) are released into the extracellular space, leading to blurred cell membrane boundaries and diffused red dye. As shown in Figure 1, H_MRGX2_RBL-2H3 cells exhibited clear degranulation after 1 h of treatment with 20 μM C48-80, PAMP-12, Substance P, and (R)-ZINC-3573.

### 3.2. Compound-Induced Increased Tryptase and β-Hexosaminidase Levels in Mast Cells and Basophilic Leukemia Cells

**Figure 2:**
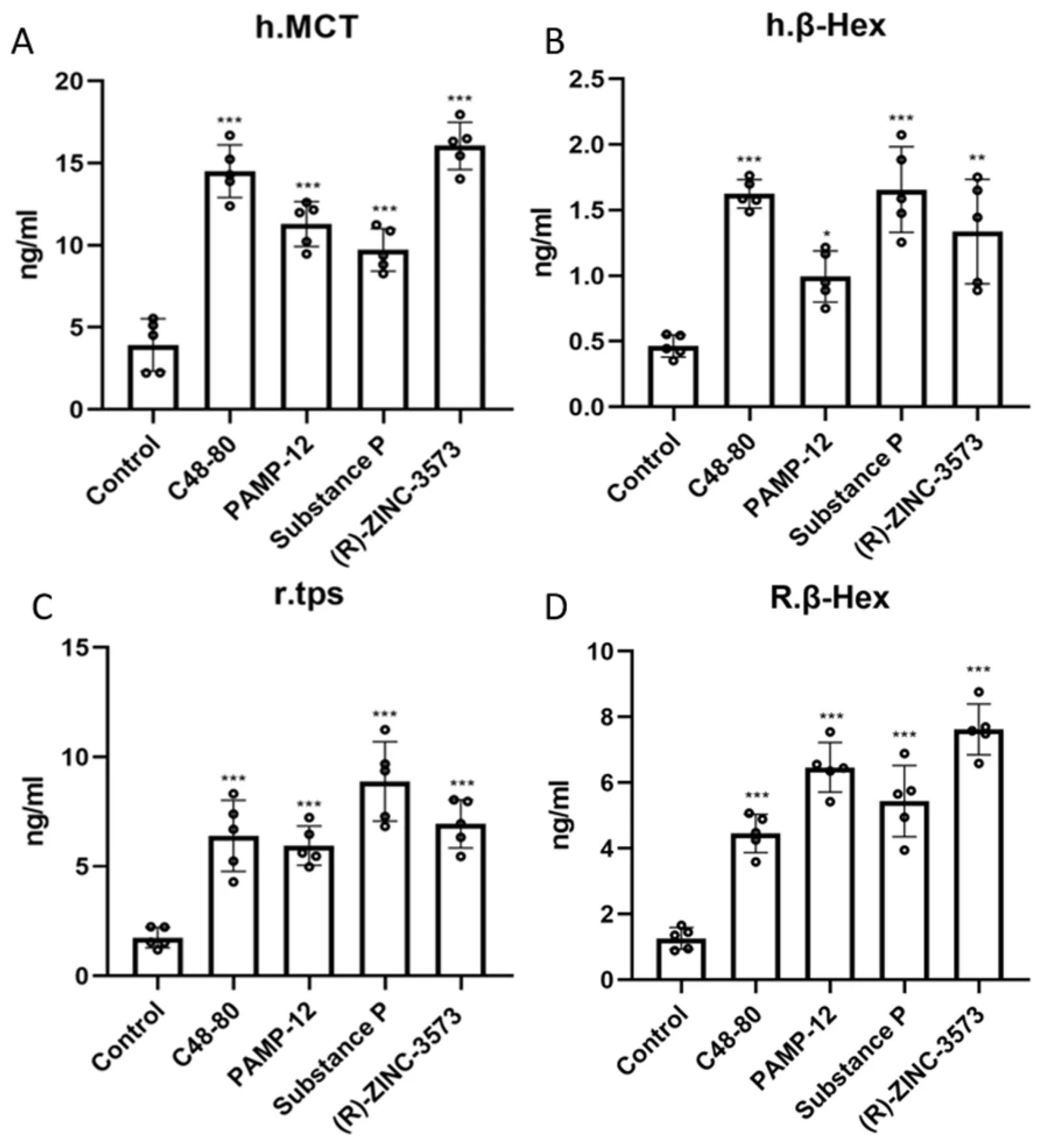
Quantification of mast cell degranulation compounds. Effects of compounds on the secretion of tryptase (**A**) (*n* = 5) and β-hexosaminidase (**B**) (*n* = 5) from H_MRGX2 HMC-1 cells. Effects of compounds on the secretion of tryptase (**C**) (*n* = 5) and β-hexosaminidase (**D**) (*n* = 5) from H_MRGX2 RBL-2H3 cells. Data are presented as means ± SD from three independent experiments. Compared with the control group, * *p* < 0.05, ** *p* < 0.01, *** *p* < 0.001 (determined by one-way ANOVA followed by Dunnett’s post hoc test).

Compared to the control group, all compounds induced varying degrees of elevation in tryptase and β-hexosaminidase in H_MRGX2 RBL-2H3 and H_MRGX2 HMC-1 cells after 1 h of intervention (Figure 2).

### 3.3. RNA-Seq Quality Analysis

Approximately 236 Gb of raw data were obtained from RNA-seq (deposited in GEO under accession GSE329896 and GSE330505). Each sample yielded over 4 × 10^7^ raw reads, with an effective read rate of 100% after quality control. Base quality analysis showed that the percentage of Q30 bases was above 97.63% (Table S1). Further quality control on alignment showed an average overall mapping rate of approximately 96.5%, with uniquely mapped reads accounting for 88.2% and multi-mapped reads for 5.3% across all samples. These mapping statistics collectively confirm the high reliability and quality of the RNA-seq data, ensuring its suitability for downstream analyses.

### 3.4. Compound-Induced Transcriptional Differences in Mast Cells and Basophilic Leukemia Cells

**Figure 3:**
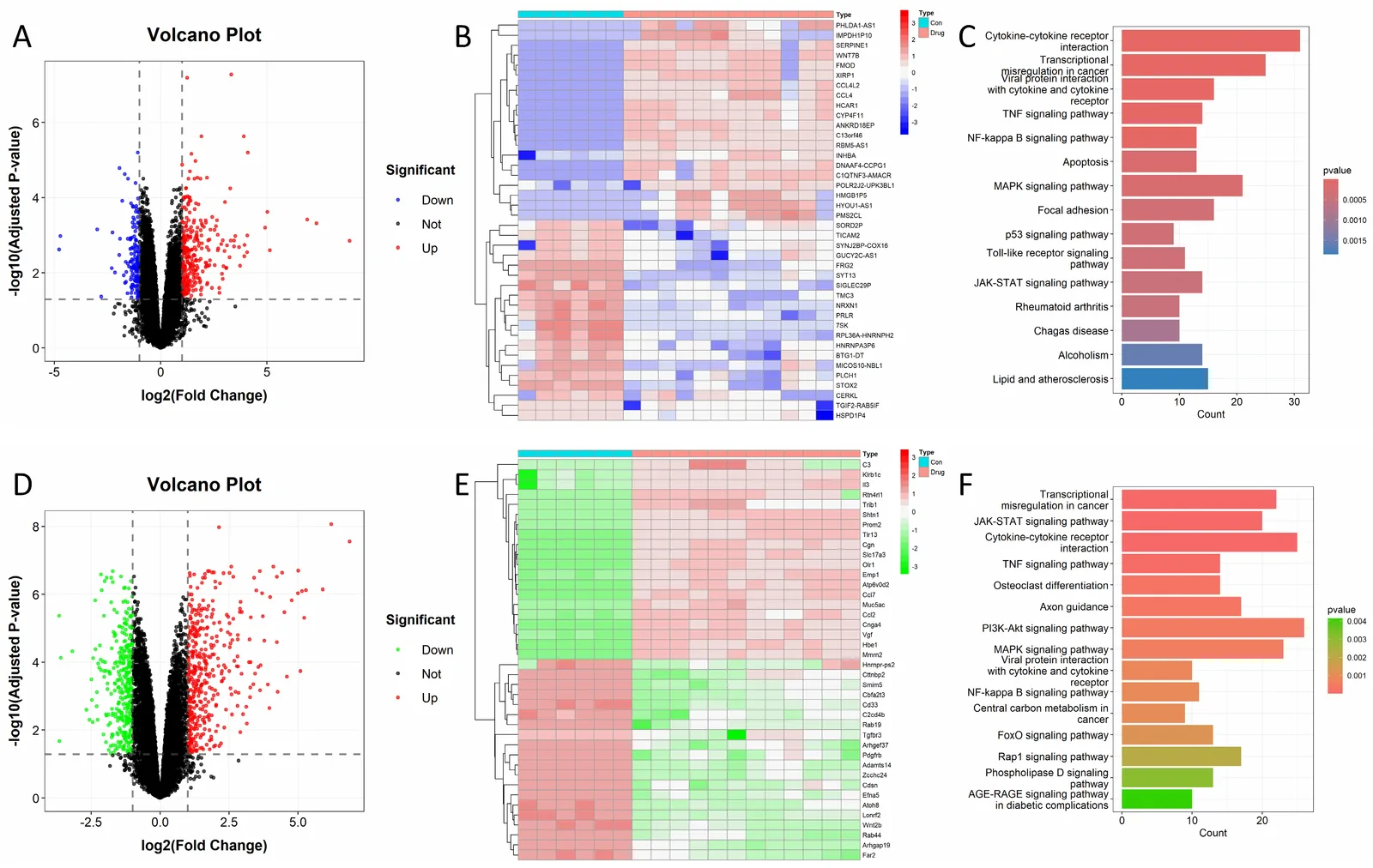
Results of differential gene expression analysis. Volcano plot (**A**) and heatmap (**B**) of DEGs in H_MRGX2_HMC−1-1 cells; Volcano plot (**D**) and heatmap (**E**) of DEGs in H_MRGX2_RBL-2H3 cells. Enriched items in KEGG pathway analysis of DEGs from H_MRGX2_HMC−1-1 cells (**C**) and H_MRGX2_RBL-2H3 cells (**F**).

Following compound intervention, a total of 712 DEGs were identified in H_MRGX2_HMC-1 cells, comprising 554 upregulated (red) and 158 downregulated (blue) genes, as displayed in the volcano plot (Figure 3A). A heatmap shows the top 20 upregulated (red) and downregulated (blue) DEGs (Figure 3B). In H_MRGX2_RBL-2H3 cells, 875 DEGs were identified (Figure 3D), with 483 upregulated (red) and 392 downregulated (green) genes. A heatmap presents the top 20 upregulated (red) and downregulated (green) DEGs (Figure 3E). The bipolar distribution pattern in the volcano plots reveals the extensive regulatory effects of pseudoallergen-positive compounds on pseudoallergy-related genes.

### 3.5. Compound-Induced Changes in Biological Processes and Downstream Pathways

In H_MRGX2_HMC-1 cells, 26 significantly enriched pathways were identified after intervention with pseudoallergen-positive compounds (Table S2), with the top 15 displayed in Figure 3C. In H_MRGX2_RBL-2H3 cells, 15 significantly enriched pathways were obtained (Table S3), presented as a bar plot (Figure 3F). Common pathways between the two cell lines included cytokine–cytokine receptor interaction, transcriptional misregulation in cancer, JAK-STAT signaling pathway, viral protein interaction with cytokine and cytokine receptor, TNF signaling pathway, MAPK signaling pathway, PI3K-Akt signaling pathway, and FoxO signaling pathway. GO analysis results for H_MRGX2_HMC-1 cells primarily revealed biological processes closely related to inflammation, immune response, and intracellular signal transduction. GO analysis results for H_MRGX2_RBL-2H3 cells included inflammation and immune response, cellular dynamic behavior, and direct processes of immune activation (Figure S1).

### 3.6. Construction of Pseudoallergic Reaction Characteristic Gene Signature

**Figure 4:**
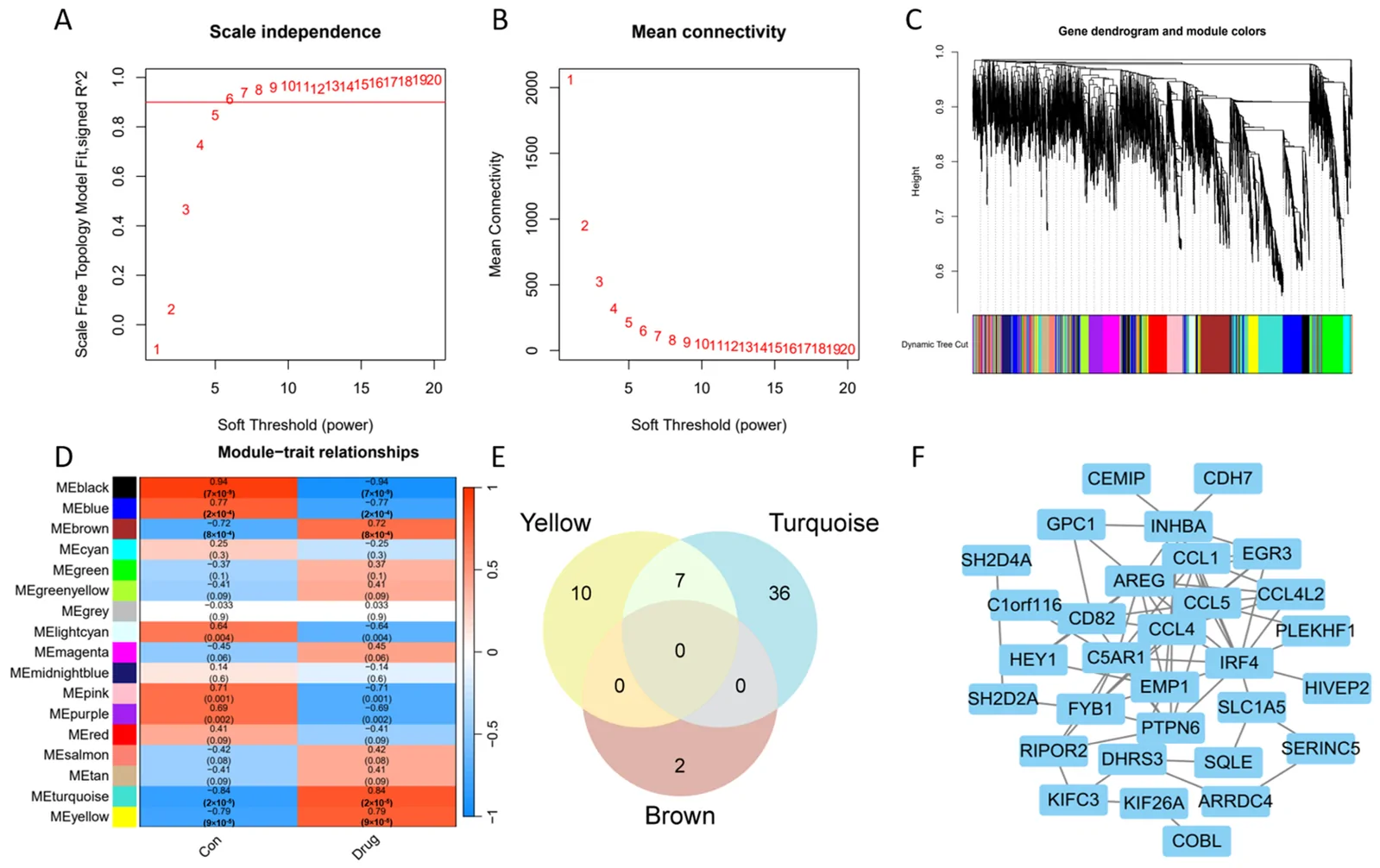
WGCNA and identification of characteristic gene modules. Soft threshold determination (**A**,**B**). The red line indicates the scale-free fitting index in WGCNA. Co-expression module construction (**C**). Module-trait association heatmap (**D**). The color intensity represents the absolute value of the correlation coefficient, with red indicating positive correlation and blue indicating negative correlation. Venn diagram (**E**), protein–protein interaction (PPI) network (**F**).

A scale-free network was constructed based on a soft threshold of 6 and a scale-free topology fit index (R^2^) of 0.85 (Figure 4A,B). Genes with similar expression patterns were clustered into modules, resulting in 17 co-expression network modules, each represented by a unique color (Figure 4C). Among these, the turquoise, yellow, and brown module eigengenes showed a significant positive correlation with pseudoallergy (r = 0.84, r = 0.79, r = 0.72, respectively; *p* < 0.05) (Figure 4D). Within these modules, candidate key genes were filtered based on |GS| > 0.9 and |MS| > 0.9, yielding 43, 17, and two genes, respectively (Figure 4E). Subsequently, a characteristic gene network comprising 31 genes was obtained by calculating topological features using the cytoHubba plugin with the MCC algorithm (Figure 4F).

### 3.7. Causal Effect of Gene Expression Levels on Drug Allergy

**Figure 5:**
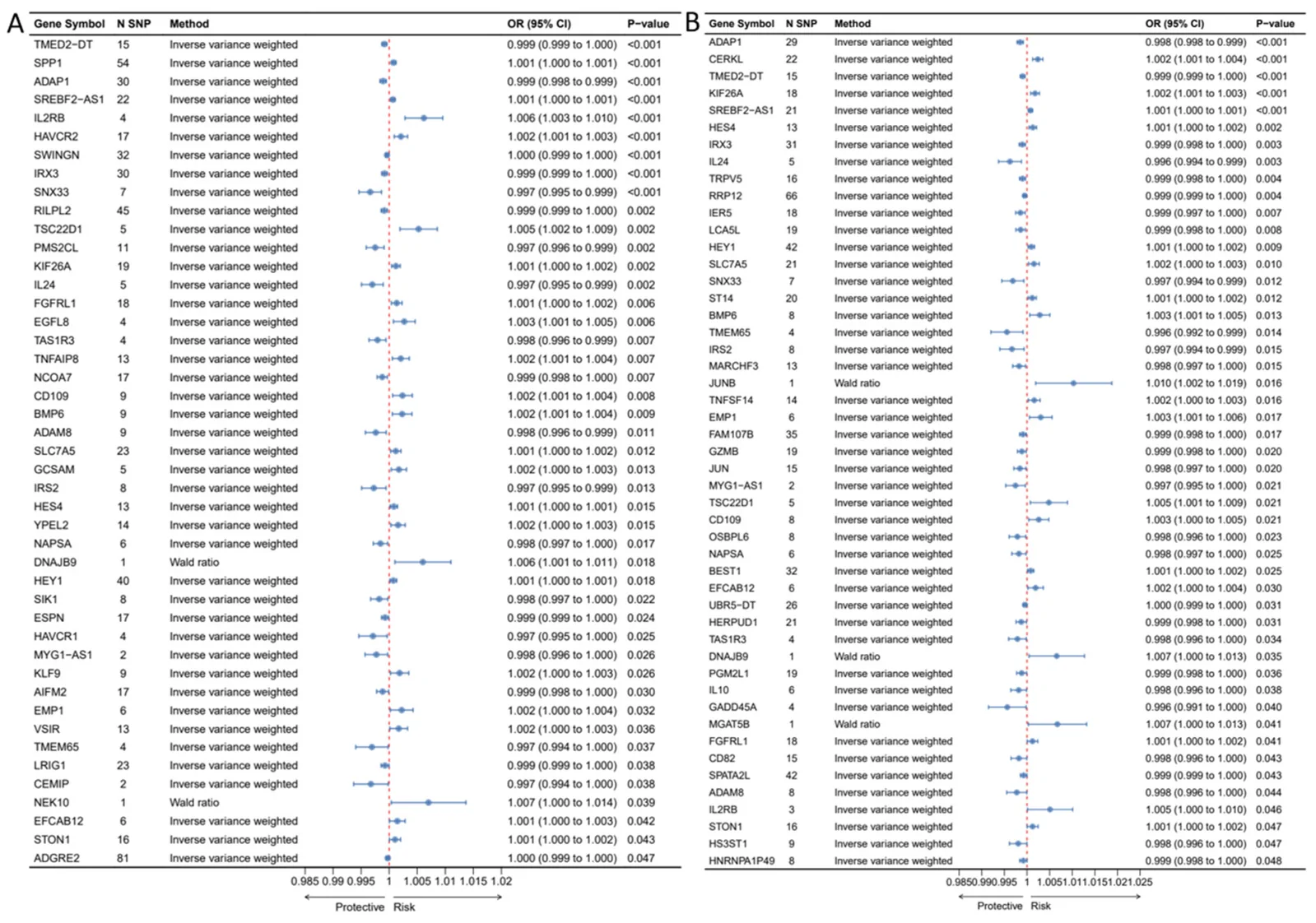

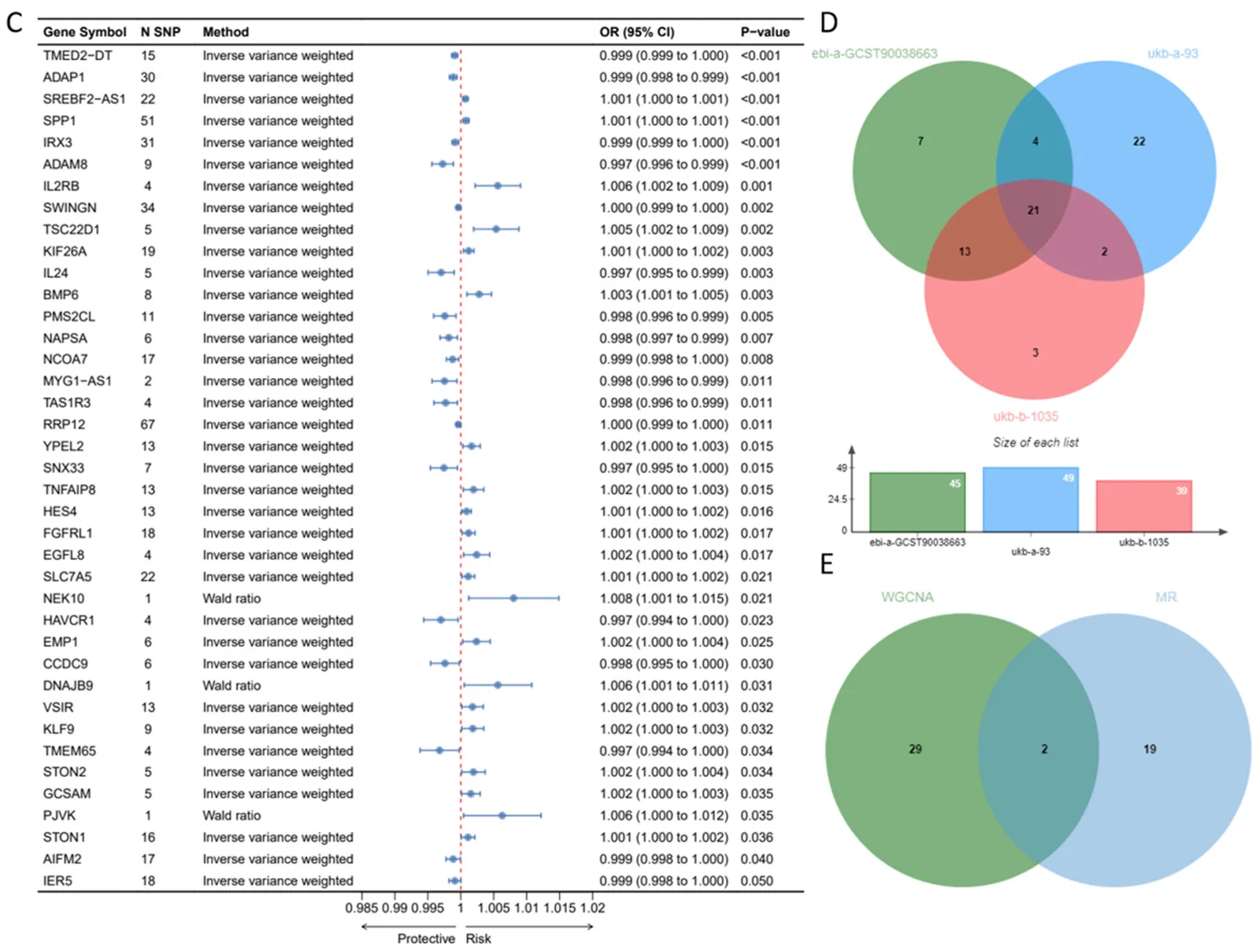
MR analysis forest plots and Venn diagrams. Causal associations of critical genes with drug allergy across different datasets: (**A**) ebi-a-GCST90038663, (**B**) ukb-b-10351, and (**C**) ukb-a-93. (**D**) Venn diagram illustrating the intersection of genes identified across the three MR datasets. (**E**) Venn diagram showing the intersection of genes identified from WGCNA of transcriptome data and MR-identified genes. *p* < 0.05 was considered statistically significant.

**Figure 6:**
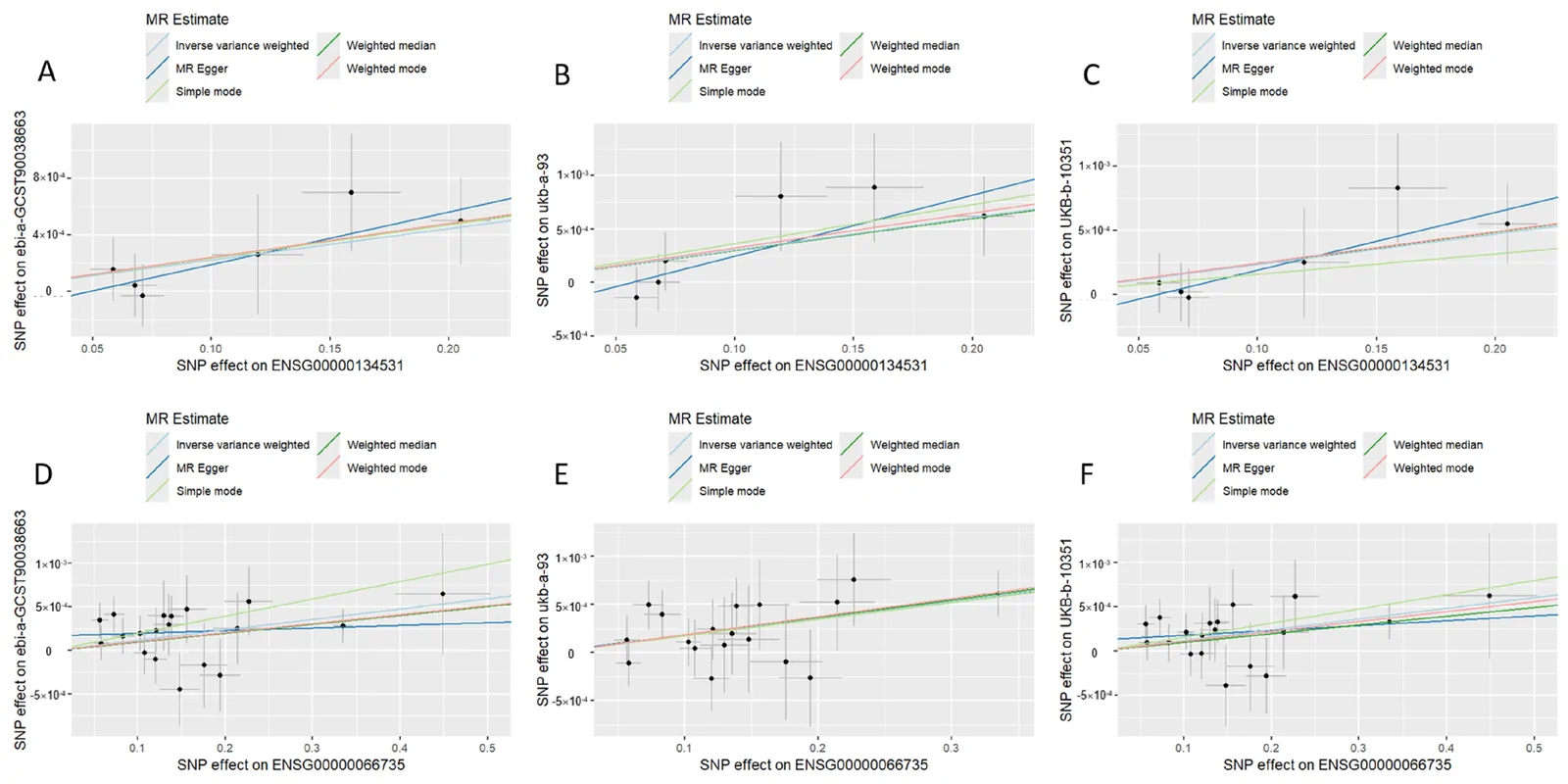
Scatter plots of MR analysis illustrating the association between marker genes and drug pseudoallergy. Scatter plots for EMP1 across datasets: (**A**) ebi-a-GCST90038663, (**B**) ukb-b-10351, and (**C**) ukb-a-93. Scatter plots for KIF26A across datasets: (**D**) ebi-a-GCST90038663, (**E**) ukb-b-10351, and (**F**) ukb-a-93.

The characteristics of the three outcome datasets obtained from the OpenGWAS database are summarized in Table 1. MR analysis identified a total of 133 genes with potential causal associations with drug allergy (Figure 5A–C). Specifically, 45 genes were identified in the ebi-a-GCST90038663 dataset, 49 in ukb-a-93, and 39 in ukb-b-10351, with 21 genes overlapping across all three datasets (Figure 5D). Among these, 11 were identified as risk genes (OR > 1), including SREBF2-AS1, IL2RB, TSC22D1, KIF26A, FGFRL1, BMP6, SLC7A5, HES4, DNAJB9, EMP1, and STON1. Conversely, 10 were identified as protective genes (OR < 1), including TMED2-DT, ADAP1, IRX3, SNX33, IL24, TAS1R3, ADAM8, NAPSA, MYG1-AS1, and TMEM65. By intersecting the causal genes identified through MR with the DEGs obtained from the WGCNA of drug allergy transcriptomic data, two key marker genes were identified (Figure 5E). The MR analysis demonstrated that increased expression levels of EMP1 which was analyzed using six instrumental SNPs in each of the three GWAS datasets, (OR = 1.002; 95% CI: 1.000–1.004; *p* = 0.025) and KIF26A analyzed using 19 in ebi-a-GCST90038663, 18 in ukb-a-93, 19 in ukb-b-10351 instrumental SNPs, (OR = 1.002; 95% CI: 1.001–1.003; *p* < 0.001) were positively correlated with the risk of drug allergy. For EMP1 and KIF26A, sensitivity analyses were statistically feasible due to the sufficient number of instrumental SNPs. These analyses consistently showed no statistically significant evidence of horizontal pleiotropy (MR-Egger intercept *p* > 0.05) or heterogeneity (Cochran’s Q *p* > 0.05) across all three outcome datasets. This indicates that the causal associations of EMP1 (Figure 6A–C) and KIF26A (Figure 6D–F) are robust and minimally influenced by outliers or pleiotropic effects. Additionally, leave-one-out analysis (Figure S2) further confirmed the stability of these findings.

### 3.8. Expression and Functional Enrichment Analysis of Marker Genes

**Figure 7:**
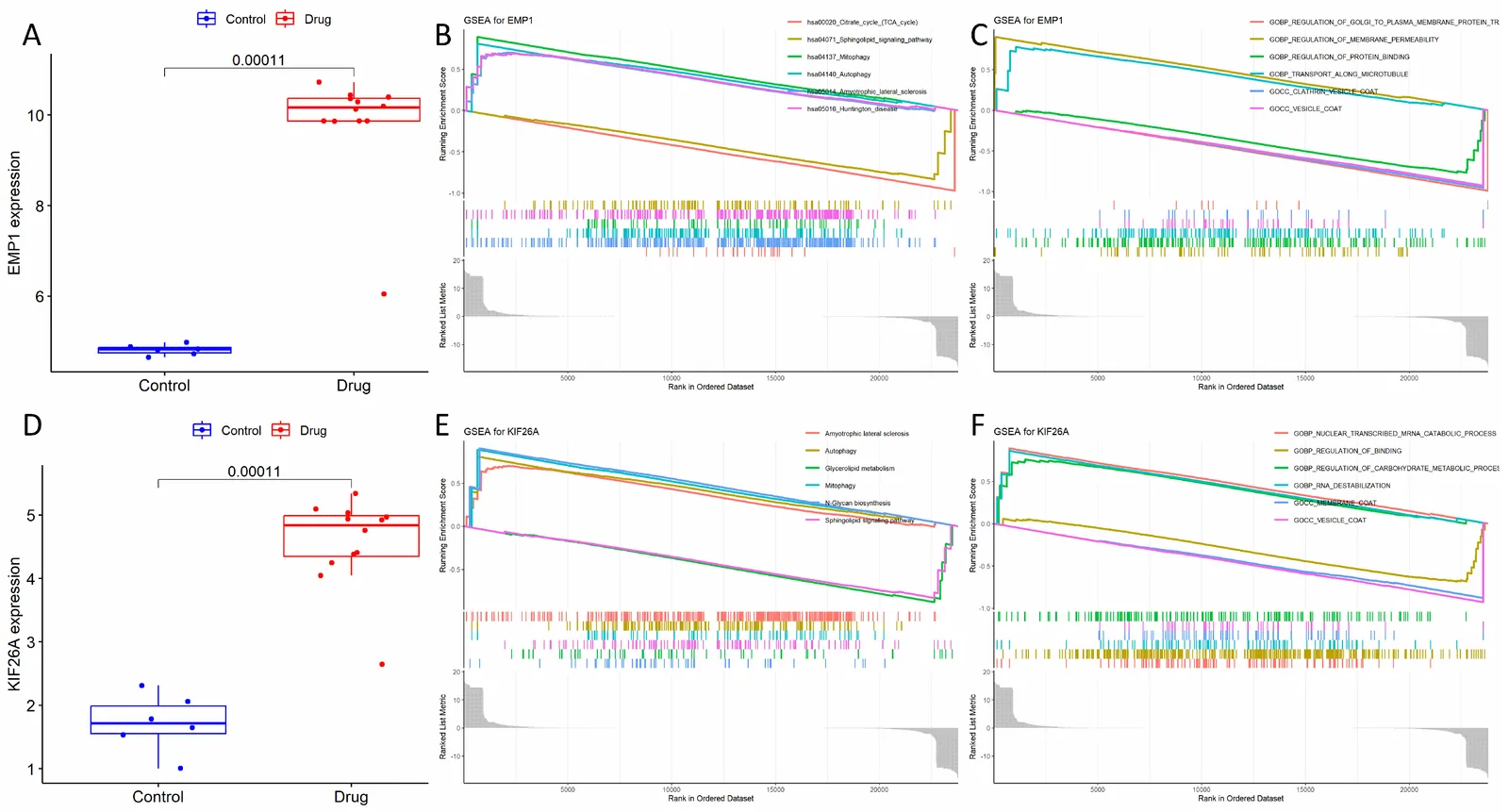
Expression and functional characterization of drug pseudoallergy marker genes. (**A**) Relative expression levels of the EMP1 gene from transcriptome data. (**B**) KEGG enrichment analysis of EMP1. (**C**) GO enrichment analysis of EMP1. (**D**) Relative expression levels of the KIF26A gene from transcriptome data. (**E**) KEGG enrichment analysis of KIF26A. (**F**) GO enrichment analysis of KIF26A.

The expression changes in the two marker genes under pseudoallergenic exposure were further confirmed within the transcriptomic data. The results showed that the drug Pseudoallergy risk genes EMP1 and KIF26A were significantly upregulated in the transcriptomic profiles (Figure 7A,D). GSEA further revealed that high expression of the EMP1 gene was significantly enriched in pathways and biological processes including sphingolipid signaling, mitophagy, autophagy, membrane transport, and vesicle formation (Figure 7B,C). Meanwhile, KIF26A was predominantly enriched in N-glycan biosynthesis, glycerolipid metabolism, and mRNA catabolic processes (Figure 7E,F). The MR leave-one-out sensitivity analysis results of EMP1 and KIF26A are presented in Figure S2.

## 4. Discussion

Pseudoallergic reactions can occur immediately upon first drug administration, posing significant challenges for prevention and risk assessment. In clinical practice, various drug classes have been reported to induce pseudoallergic reactions, including antibiotics (e.g., quinolones, β-lactams) [14], muscle relaxants [15], contrast agents [16], and TCMIs [17]. Clinical manifestations range from skin flushing, urticaria, and angioedema to bronchospasm and dyspnea, with severe cases progressing to anaphylactic shock and even death [18]. Currently, data on drug-induced pseudoallergic reactions are relatively scarce. While clinical case reports are abundant, systematic molecular mechanism studies, particularly those based on high-throughput omics technologies and in vitro/in vivo experimental data, are extremely limited. This study, for the first time, systematically constructed a cell degranulation transcriptome database induced by various known pseudoallergen-positive compounds, filling a crucial gap in this field.

The selection of four pseudoallergen-positive control compounds—C48-80 [19], PAMP-12 [20], Substance P [21], and (R)-ZINC-3573 [22]—was based on their ability to represent diverse mechanisms by which different compound types induce pseudoallergy. This selection laid the foundation for establishing a widely representative characteristic gene signature. The neutral red staining and ELISA experiment in this study visually confirmed the biological activity of the selected positive compounds, with cells showing clear degranulation after 1 h of treatment. The high quality of the obtained transcriptome sequencing data ensured the reliability of subsequent bioinformatics analyses. Differences in the number and distribution of DEGs between the two cell lines may reflect species-specific variations in transcriptional regulatory networks and signal transduction mechanisms, highlighting the need to consider species differences in drug safety evaluation. KEGG and GO enrichment analyses revealed that the core signaling networks activated in pseudoallergic reactions are significantly enriched in pathways related to inflammation and immunity (cytokine–cytokine receptor interaction, TNF signaling pathway), intracellular signaling pathways (MAPK, JAK-STAT, PI3K-Akt signaling pathway), and cellular stress and transcriptional regulation (FoxO signaling pathway, transcriptional misregulation in cancer). Although both models demonstrated conserved core pathways upon pseudoallergen exposure, subtle discrepancies in downstream transcriptional regulatory networks might exist due to species differences. Furthermore, our transcriptome analysis focused solely on a single time point (1 h) after the compound treatment. Although this time point is crucial for capturing the rapid onset of pseudoallergic reactions, it may not have provided a comprehensive understanding of the complete temporal dynamics of transcriptional responses during the later stages or resolution of pseudoallergy. Collectively, these pathways depict a comprehensive molecular landscape of mast cells in pseudoallergic reactions, from sensing stimuli and signal transduction to inflammatory mediator release and cell fate regulation.

Given the relatively smaller sample size of our in vitro study compared to large-scale clinical cohort studies, weighted gene co-expression network analysis (WGCNA) [23] was chosen for characteristic gene screening using human H_MRGX2_HMC-1 cell transcriptome data. The approach for WGCNA, including the selection of a robust soft-thresholding power, signed network type, and a substantial minModuleSize, was designed to maximize network stability and module reliability within the constraints of our controlled in vitro experimental design, where biological replicates are considered adequate for identifying treatment-induced transcriptional changes. The genes identified in the pseudoallergy-specific gene signature encompass multiple facets critical to immune response: complement activation (C5AR1) [24], cytokine signaling (LIF, IRF4) [25,26], chemokine networks (CCL4, CCL5) [27], endoplasmic reticulum stress (XBP1) [28], vesicle transport (EMP1, KIF26A) [29], and signal regulation (PTPN6, FYB1) [30]. These genes form the core network nodes of the pseudoallergic characteristic gene signature. Because current publicly available GWAS summary statistics broadly categorize phenotypes as ‘drug allergy’ or ‘anaphylactic reactions,’ these datasets inherently conflate IgE-mediated Type I hypersensitivity and non-IgE-mediated pseudoallergic reactions. Consequently, following transcriptomic analysis, the intersection of key genes identified by pseudoallergy-specific WGCNA with causally inferred genes from our MR analysis yielded two potential high-risk pseudoallergy marker genes: EMP1 and KIF26A. It is important to acknowledge that an OR close to 1 indicates a subtle effect at the individual genetic variant level. In the context of polygenic complex traits and molecular cis-eQTL MR analyses, individual gene expression instruments often exhibit modest effect sizes. While these subtle effects may not directly translate to immediate actionable clinical utility for individual diagnosis, they are crucial for uncovering underlying biological pathways (such as vesicle transport and membrane coating) that cumulatively modulate individual susceptibility. These findings suggest that genetic variations impacting EMP1 and KIF26A expression contribute to a complex polygenic background, influencing the overall risk.

EMP1, encoding Epithelial Membrane Protein 1, is a transmembrane glycoprotein localized to the plasma membrane and intracellular membrane-bound organelles, implicated in apoptosis and follicular assembly [31]. KIF26A encodes an atypical kinesin, primarily functioning as a molecular motor transporting intracellular vesicles and organelles along microtubules [32]. Both EMP1 and KIF26A exhibited significantly higher expression in the drug-treated group, and their upregulation was significantly enriched in pathways and functions related to inflammation, cellular metabolic reprogramming, membrane transport, and cellular autophagy. Elevated expression or enhanced function of these genes at the genetic level, by mediating alterations in these biological pathways, contributes to an increased causal risk of drug-induced pseudoallergy.

## 5. Conclusions

In summary, this comprehensive study utilized high-throughput RNA sequencing to systematically characterize the transcriptomic profiles of mast cell degranulation induced by pseudoallergy-positive compounds. We successfully identified and elucidated core signaling pathways, including cytokine signaling, MAPK, JAK-STAT, TNF, and PI3K-Akt pathways, and constructed a biologically significant characteristic gene network. To further validate these findings and to provide a more dynamic understanding of protein activation, future research will integrate proteomics and phosphoproteomics analyses, particularly focusing on the actual activation states of the identified MAPK, JAK-STAT, and PI3K-Akt pathways.

Furthermore, by leveraging publicly available GWAS data and employing MR analysis, we identified EMP1 and KIF26A as potential causal risk marker genes associated with drug pseudoallergy. Our findings suggest that these two genes may play synergistic roles within “Non-IgE-mediated pathways,” specifically implicating intracellular substance transport processes in augmenting an individual’s susceptibility to drug-induced pseudoallergy. Subsequent in-depth functional validation studies, such as gene knockout or knockdown experiments of EMP1 and KIF26A in relevant cell models or in vivo systems, are imperative to definitively confirm their causal roles and dissect their underlying mechanisms in pseudoallergic reactions.

However, certain limitations warrant consideration. During data acquisition, the unavailability of dedicated GWAS datasets specifically for pseudoallergy necessitated the cautious inclusion of broader anaphylactic reaction datasets, encompassing both pseudoallergic and IgE-mediated allergic reactions. Consequently, the identified pseudoallergy risk genes represent an intersection of these GWAS findings and our in vitro model data. Another limitation pertains to the tissue source of the eQTL exposure data. While eQTLGen provides robust cis-eQTLs, these are derived from whole blood, which may not perfectly reflect the tissue-specific gene expression regulation within mast cells or basophils. Future studies using mast cell-specific eQTL data, once available, would provide more precise insights. While some effect sizes derived from MR analysis were relatively modest, their biological significance in complex disease susceptibility studies remains noteworthy. Moreover, this study primarily relied on human- and rat-transformed cell lines. Future investigations should endeavor to validate these findings using primary human mast cells or basophils isolated from individuals with clinical pseudoallergy, thereby enhancing physiological relevance and clinical translatability.

Collectively, this research not only addresses a significant void in transcriptomic data pertaining to pseudoallergic reactions but also pioneers novel avenues and provides valuable tools for drug safety evaluation and the development of anti-allergic therapeutics. It underscores substantial scientific value and holds promising clinical application prospects. Future endeavors will focus on robustly validating these discoveries and facilitating their translation into clinical utility.

## Figures and Tables

**Table 1:** Data summary.

Traits	ID	Sample Size	nsnp	Ancestry
Drug allergy or anaphylactic reaction	ebi-a-GCST90038663	484,598	9,587,836	European
Allergy or anaphylactic reaction to drug	ukb-b-10351	462,933	9,851,867	European
Allergy or anaphylactic reaction to drug	ukb-a-93	337,159	10,894,596	European

## Data Availability

The RNA-seq data generated in this study have been deposited in the Gene Expression Omnibus (GEO) database under accession numbers GSE329896 and GSE330505. All other data generated or analyzed during this study are included in this published article and its Supplementary Information files.

## Supplementary Materials

The following supporting information can be downloaded at https://www.mdpi.com/article/10.3390/cimb48101000/s1.

## Abbreviations

The following abbreviations are used in this manuscript:

bp	Base-pair
cis-eQTLs	Cis-expression quantitative trait loci
cpm	Count-per-million
DEG	Differentially expressed gene
FDR	False discovery rate
GLM	Generalized linear models
GO	Gene Ontology
GSEA	Gene set enrichment analysis
GWAS	Genome-wide association study
IVW	Inverse-variance weighted
KEGG	Kyoto Encyclopedia of Genes and Genomes
MR	Mendelian randomization
NES	Normalized enrichment scores
OR	Odds ratio
PCA	Principal component analysis
RNA-seq	RNA sequencing
SNPs	Single nucleotide polymorphisms
TOM	Topological overlap matrix
WGCNA	Weighted gene co-expression network analysis
