# Supplementary material for: Identification of Key Genetic Markers for Drug Pseudoallergy via Experimental Transcriptomics and GWAS-Based Causal Inference
Source: Curr Issues Mol Biol. 2026 Sep 29;48(10):1000. doi: 10.3390/cimb48101000 (PMC13703276; doi:10.3390/cimb48101000)
Supplement: Supplementary file 1 [file cimb-48-01000-s001.zip › cimb-4563546-supplementary.pdf]

# Supplementary Materials for

# Identification of Key Genetic Markers for Drug Pseudoallergy via Experimental Transcriptomics and GWAS-based Causal Inference

**Supplementary Table S1: Sequencing Data Statistics Table**

| SamSamplep le | Raw reads | Raw bases | Clean reads | Clean bases | Error rate(%) | Q20(%) | Q30(%) | GC content(%) |
|---|---|---|---|---|---|---|---|---|
| HK1 | 44310960 | 6646644000 | 44264780 | 6618627070 | 0.0087 | 99.29 | 98.23 | 48.54 |
| HK2 | 40337242 | 6050586300 | 40290974 | 6019227776 | 0.0089 | 99.16 | 97.91 | 51.36 |
| HK3 | 47027968 | 7054195200 | 46981148 | 7023262142 | 0.0089 | 99.09 | 97.72 | 50.55 |
| HK4 | 44300040 | 6645006000 | 44258474 | 6613778835 | 0.0089 | 99.1 | 97.75 | 49.27 |
| HK5 | 48969910 | 7345486500 | 48921394 | 7306400007 | 0.0089 | 99.11 | 97.77 | 49.06 |
| HK6 | 40097704 | 6014655600 | 40057914 | 5980148530 | 0.0086 | 99.37 | 98.42 | 45.63 |
| H1_1 | 46753378 | 7013006700 | 46705584 | 6981741264 | 0.0088 | 99.2 | 97.99 | 48.19 |
| H1_2 | 39212712 | 5881906800 | 39170010 | 5852021558 | 0.0088 | 99.19 | 97.97 | 50.4 |
| H1_3 | 41503932 | 6225589800 | 41460740 | 6196930554 | 0.0087 | 99.26 | 98.14 | 49.51 |
| H2_1 | 44242930 | 6636439500 | 44199982 | 6607566864 | 0.0089 | 99.1 | 97.75 | 49.41 |
| H2_2 | 46892976 | 7033946400 | 46848540 | 7001253005 | 0.0089 | 99.1 | 97.75 | 49.91 |
| H2_3 | 42614540 | 6392181000 | 42569874 | 6367213958 | 0.0089 | 99.12 | 97.79 | 49.69 |
| H3_1 | 36642062 | 5496309300 | 36598952 | 5468830694 | 0.0088 | 99.17 | 97.94 | 49.6 |

| | | | | | | | | |
|---|---|---|---|---|---|---|---|---|
| H3_2 | 42965134 | 6444770100 | 42925770 | 6414045072 | 0.0089 | 99.1 | 97.76 | 48.87 |
| H3_3 | 43594432 | 6539164800 | 43551628 | 6506566222 | 0.0089 | 99.1 | 97.76 | 49.01 |
| H4_1 | 46477846 | 6971676900 | 46435518 | 6933581772 | 0.0089 | 99.13 | 97.83 | 48.95 |
| H4_2 | 38715912 | 5807386800 | 38680488 | 5778333502 | 0.009 | 99.05 | 97.63 | 48.79 |
| H4_3 | 49332418 | 7399862700 | 49286498 | 7370051149 | 0.0088 | 99.23 | 98.06 | 48.73 |
| RK1 | 45429666 | 6814449900 | 45380732 | 6781892676 | 0.0089 | 99.13 | 97.81 | 50.01 |
| RK2 | 41062806 | 6159420900 | 41015558 | 6132888442 | 0.0088 | 99.19 | 97.96 | 50.61 |
| RK3 | 37256288 | 5588443200 | 37211872 | 5564265513 | 0.0087 | 99.27 | 98.18 | 49.64 |
| RK4 | 46806624 | 7020993600 | 46720882 | 6986510544 | 0.0091 | 98.99 | 97.41 | 49.34 |
| RK5 | 46509832 | 6976474800 | 46461338 | 6946930242 | 0.0089 | 99.15 | 97.87 | 49.77 |
| RK6 | 38953840 | 5843076000 | 38909144 | 5817838941 | 0.0088 | 99.2 | 98.01 | 49.42 |
| R1_1 | 49737708 | 7460656200 | 49687568 | 7427243017 | 0.0089 | 99.15 | 97.87 | 49.5 |
| R1_2 | 50016944 | 7502541600 | 49927154 | 7463676408 | 0.0091 | 99 | 97.44 | 49.49 |
| R1_3 | 48812720 | 7321908000 | 48764046 | 7290480515 | 0.0089 | 99.16 | 97.91 | 49.2 |
| R2_1 | 41828572 | 6274285800 | 41777268 | 6239298326 | 0.0088 | 99.22 | 98.04 | 49.51 |
| R2_2 | 44008120 | 6601218000 | 43924530 | 6565784789 | 0.0091 | 99 | 97.45 | 49.49 |
| R2_3 | 45302462 | 6795369300 | 45256004 | 6768021320 | 0.0089 | 99.13 | 97.82 | 49.64 |

| | | | | | | | | |
|---|---|---|---|---|---|---|---|---|
| R3_1 | 44313008 | 6646951200 | 44264484 | 6613479943 | 0.0088 | 99.17 | 97.91 | 49.62 |
| R3_2 | 45997060 | 6899559000 | 45913990 | 6861860094 | 0.0091 | 98.98 | 97.39 | 49.44 |
| R3_3 | 45389070 | 6808360500 | 45342238 | 6774644640 | 0.0089 | 99.16 | 97.91 | 49.42 |
| R4_1 | 45929276 | 6889391400 | 45884790 | 6862302924 | 0.0089 | 99.13 | 97.83 | 49.73 |
| R4_2 | 37364054 | 5604608100 | 37322508 | 5582602220 | 0.0088 | 99.24 | 98.1 | 49.69 |
| R4_3 | 43284234 | 6492635100 | 43237674 | 6464933900 | 0.0087 | 99.28 | 98.19 | 49.56 |

**Supplementary Table S2: KEGG enrichment analysis results of DEGs in HMC-1 cells**

| Description | GeneRatio | BgRatio | pvalue | p.adjust | qvalue |
|---|---|---|---|---|---|
| Cytokine-cytokine receptor interaction | 31/277 | 298/9376 | 8.06632166130045e-10 | 1.33272128936058e-07 | 1.16310221616923e-07 |
| Transcriptional misregulation in cancer | 25/277 | 201/9376 | 9.69251846807692e-10 | 1.33272128936058e-07 | 1.16310221616923e-07 |
| Viral protein interaction with cytokine and cytokine receptor | 16/277 | 100/9376 | 3.16267376407711e-08 | 2.89911761707069e-06 | 2.53013901126169e-06 |
| TNF signaling pathway | 14/277 | 119/9376 | 1.01894751114455e-05 | 0.000665840186329947 | 0.000581096889887954 |
| NF-kappa B signaling pathway | 13/277 | 105/9376 | 1.2106185205999e-05 | 0.000665840186329947 | 0.000581096889887954 |
| Apoptosis | 13/277 | 137/9376 | 0.000199561080652573 | 0.00841518403574772 | 0.00734416061301619 |
| MAPK signaling pathway | 21/277 | 300/9376 | 0.000214204684546306 | 0.00841518403574772 | 0.00734416061301619 |
| Focal adhesion | 16/277 | 203/9376 | 0.000331161267374084 | 0.00993436530409586 | 0.00866999153812002 |
| p53 signaling pathway | 9/277 | 75/9376 | 0.000342669585260937 | 0.00993436530409586 | 0.00866999153812002 |
| Toll-like receptor signaling pathway | 11/277 | 109/9376 | 0.000361249647421668 | 0.00993436530409586 | 0.00866999153812002 |
| JAK-STAT signaling pathway | 14/277 | 168/9376 | 0.000443489150214555 | 0.0108892549697081 | 0.00950334979174522 |
| Rheumatoid arthritis | 10/277 | 95/9376 | 0.000475167489587261 | 0.0108892549697081 | 0.00950334979174522 |
| Chagas disease | 10/277 | 103/9376 | 0.000902051054035086 | 0.019081849219973 | 0.0166532502283401 |
| Alcoholism | 14/277 | 191/9376 | 0.00156405133490305 | 0.0307224369355956 | 0.026812308598338 |
| Lipid and atherosclerosis | 15/277 | 216/9376 | 0.00184182721 | 0.0337668323 | 0.0294692354 |

| | | | | | |
|---|---|---|---|---|---|
| | | | 687494 | 09374 | 699991 |
| PI3K-Akt signaling pathway | 21/277 | 362/9376 | 0.00241195042811443 | 0.0414553979832167 | 0.0361792564217164 |
| Hematopoietic cell lineage | 9/277 | 100/9376 | 0.00273458052956546 | 0.0442358615076766 | 0.0386058427703359 |
| Viral carcinogenesis | 14/277 | 205/9376 | 0.0030176820849743 | 0.0461034762982185 | 0.0402357611329907 |
| Systemic lupus erythematosus | 11/277 | 143/9376 | 0.00336357553877468 | 0.0486833301664757 | 0.0424872699634697 |
| Breast cancer | 11/277 | 148/9376 | 0.00437307600025361 | 0.0589925306063447 | 0.0514843903473554 |
| ECM-receptor interaction | 8/277 | 89/9376 | 0.00468023314810825 | 0.0589925306063447 | 0.0514843903473554 |
| Chemokine signaling pathway | 13/277 | 193/9376 | 0.00471940244850758 | 0.0589925306063447 | 0.0514843903473554 |
| Neutrophil extracellular trap formation | 13/277 | 195/9376 | 0.00514440314661354 | 0.0615091680573358 | 0.0536807284864022 |
| Melanoma | 7/277 | 73/9376 | 0.00561978525068765 | 0.0643933726641293 | 0.0561978525068765 |
| FoxO signaling pathway | 10/277 | 133/9376 | 0.00596171947050523 | 0.0655789141755576 | 0.0572325069168502 |
| IL-17 signaling pathway | 8/277 | 94/9376 | 0.00650348811877858 | 0.0687868935640042 | 0.0600321980194946 |

**Supplementary Table S3: KEGG enrichment analysis results of DEGs in H_MRGX2_RBL-2H3 cells**

| Description | GeneRatio | BgRatio | pvalue | p.adjust | qvalue |
|---|---|---|---|---|---|
| Transcriptional misregulation in cancer | 22/404 | 194/11246 | 1.75311785210484e-06 | 0.000315875429459181 | 0.000267492350857809 |
| JAK-STAT signaling pathway | 20/404 | 166/11246 | 2.02484249653321e-06 | 0.000315875429459181 | 0.000267492350857809 |
| Cytokine-cytokine receptor interaction | 25/404 | 274/11246 | 1.77509310284004e-05 | 0.00184609682695364 | 0.00156332760986965 |
| TNF signaling pathway | 14/404 | 119/11246 | 9.04966374913786e-05 | 0.00705873772432753 | 0.00597754105535159 |
| Osteoclast differentiation | 14/404 | 132/11246 | 0.000274485893767174 | 0.0171279197710716 | 0.014504412491697 |
| Axon guidance | 17/404 | 184/11246 | 0.000336796041389701 | 0.0175133941522645 | 0.0148308432261079 |
| PI3K-Akt signaling pathway | 26/404 | 356/11246 | 0.000475291037161246 | 0.0211844005134727 | 0.0179395564402215 |
| MAPK signaling pathway | 23/404 | 302/11246 | 0.000561565424858021 | 0.0219010515694628 | 0.018546437057811 |
| Viral protein interaction with cytokine and cytokine receptor | 10/404 | 84/11246 | 0.000823312305872386 | 0.0267740964866314 | 0.0226730709114186 |
| NF-kappa B signaling pathway | 11/404 | 100/11246 | 0.000898696673537274 | 0.0267740964866314 | 0.0226730709114186 |
| Central carbon metabolism in cancer | 9/404 | 71/11246 | 0.000943958529977388 | 0.0267740964866314 | 0.0226730709114186 |

| | | | | | |
|---|---|---|---|---|---|
| FoxO signaling pathway | 13/404 | 136/11246 | 0.00120559990892834 | 0.0313455976321369 | 0.0265443488720187 |
| Rap1 signaling pathway | 17/404 | 217/11246 | 0.00213058306895532 | 0.0511339936549277 | 0.0433017287698611 |
| Phospholipase D signaling pathway | 13/404 | 151/11246 | 0.00307845464389651 | 0.0686055606354079 | 0.0580971515502273 |
| AGE-RAGE signaling pathway in diabetic complications | 10/404 | 104/11246 | 0.00412996188864389 | 0.0859032072837928 | 0.0727452936175169 |

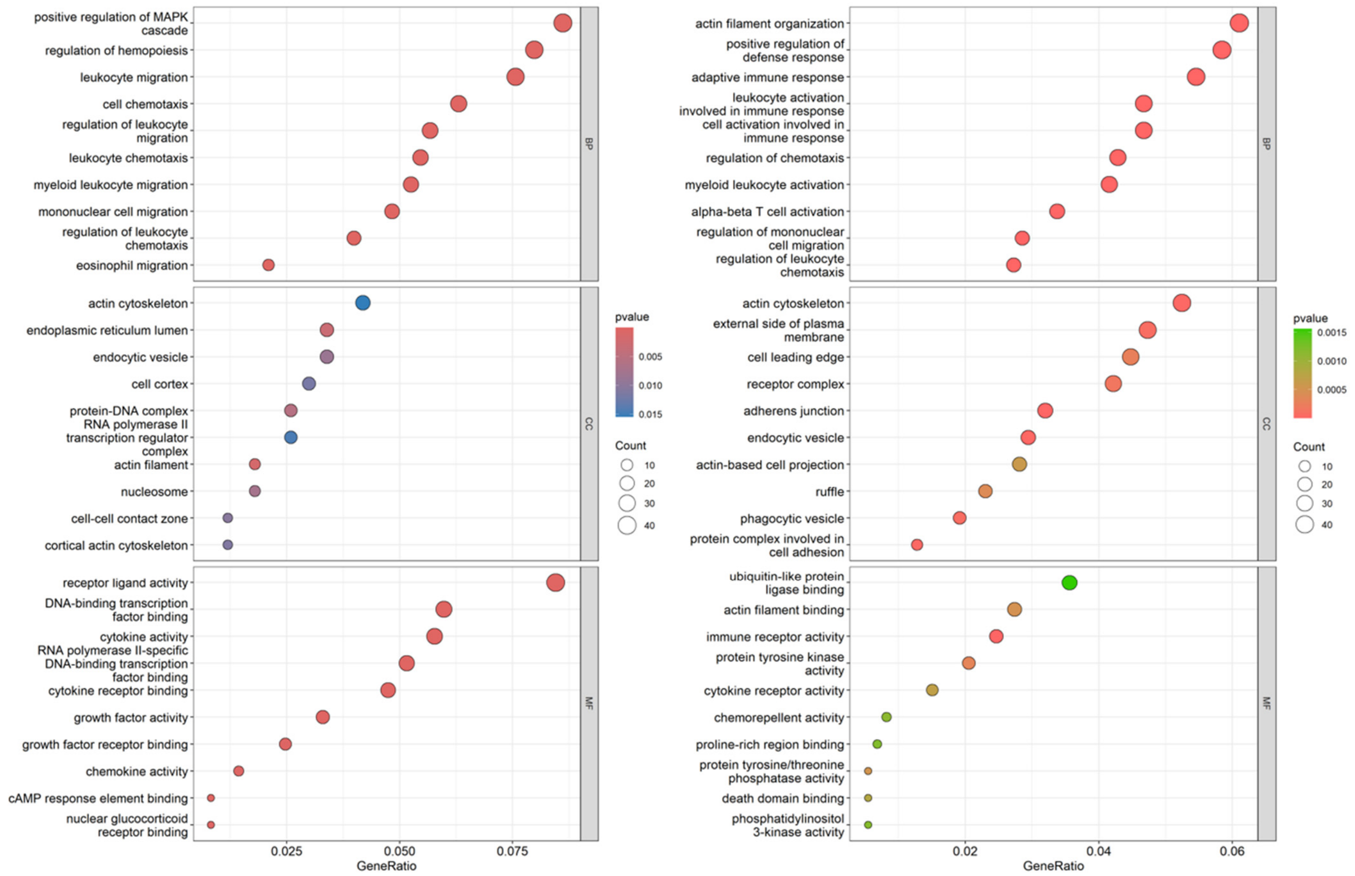


**Supplementary Figure S1: GO functional enrichment. The size of the dots in the figure represents the number of enriched genes; the smaller the P value, the redder the color of the bubble plot becomes.**

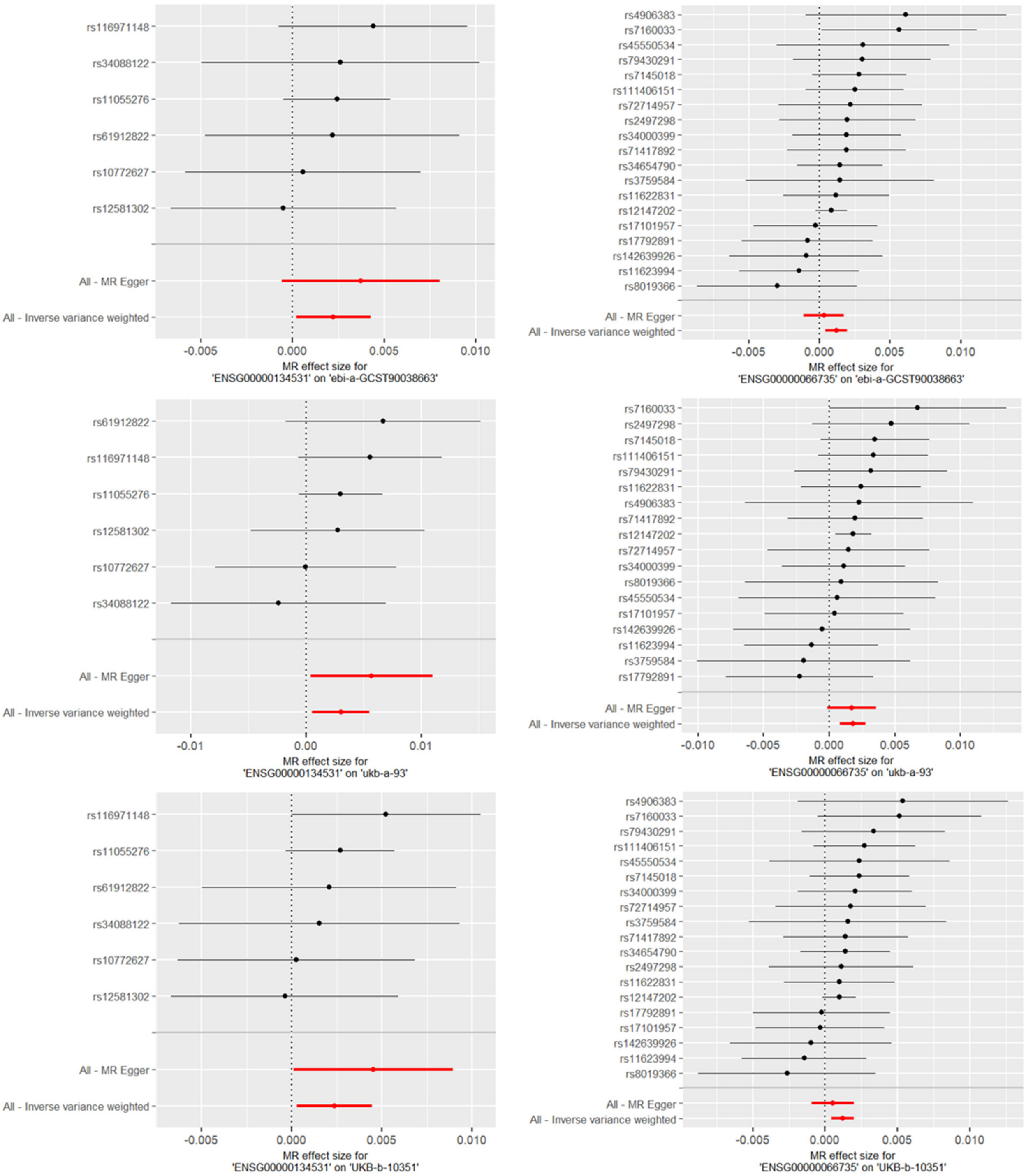


**Supplementary Figure S2: The results of MR leave-one-out sensitivity analysis of EMP1 and KIF26A**
